# G protein inhibitory α subunit 2 is a molecular oncotarget of human glioma

**DOI:** 10.7150/ijbs.79355

**Published:** 2023-01-09

**Authors:** Yin Wang, Fang Liu, Jiang Wu, Mei-qing Zhang, Jin-long Chai, Cong Cao

**Affiliations:** 1 Institute of Neuroscience, Soochow University, Institute for Excellence in Clinical Medicine of Kunshan First People's Hospital and Soochow University, Suzhou, China.; 2 Clinical Research Center of Neurological Disease, The Second Affiliated Hospital of Soochow University, Jiangsu Key Laboratory of Neuropsychiatric Diseases and Institute of Neuroscience, Soochow University, Suzhou, China.; 3 Department of Neurosurgery, The affiliated Changzhou No.2 People's Hospital of Nanjing Medical University, Changzhou, China.; 4 Department of Neurosurgery, the First Affiliated Hospital of Soochow University, Suzhou, China.; 5 The Affiliated Eye Hospital, Nanjing Medical University, Nanjing, China.

## Abstract

Identification of novel therapeutic oncotargets for human glioma is extremely important. Here we tested expression, potential functions and underlying mechanisms of G protein inhibitory α subunit 2 (Gαi2) in glioma. Bioinformatics analyses revealed that *Gαi2* expression is significantly elevated in human glioma, correlating with poor patients' survival, higher tumor grade and wild-type IDH status. Moreover, increased Gαi2 expression was also in local glioma tissues and different glioma cells. In primary and immortalized (A172) glioma cells, Gαi2 shRNA or knockout (KO, by Cas9-sgRNA) potently suppressed viability, proliferation, and mobility, and induced apoptosis. Ectopic Gαi2 overexpression, using a lentiviral construct, further augmented malignant behaviors in glioma cells. p65 phosphorylation, NFκB activity and expression of NFκB pathway genes were decreased in Gαi2-depleted primary glioma cells, but increased following Gαi2 overexpression. There was an increased binding between *Gαi2* promoter and Sp1 (specificity protein 1) transcription factor in glioma tissues and different glioma cells. In primary glioma cells *Gαi2* expression was significantly reduced following Sp1 silencing, KO or inhibition. *In vivo* studies revealed that Gαi2 shRNA-expressing AAV intratumoral injection hindered growth of subcutaneous glioma xenografts in nude mice. Moreover, Gαi2 KO inhibited intracranial glioma xenograft in nude mice. Gαi2 depletion, NFκB inhibition and apoptosis induction were observed in subcutaneous and intracranial glioma xenografts with Gαi2 depletion. Together, overexpressed Gαi2 is important for glioma cell growth possibly by promoting NFκB cascade activation.

## Introduction

Current traditional treatments have very limited effects on improving survival of glioblastoma (GBM) and other high-grade glioma (HGG) 1-3. Multiple signaling cascades are dysregulated in glioma, including EGFR, the type III mutations, VEGFR and CDK, which are essential for the uncontrolled glioma growth and malignant progression 4-8. Targeted therapies for specific molecules on the signaling pathways have become the research hotspot for glioma 4-6, 9.

G protein inhibitory α subunits (Gαi proteins) have three members, Gαi1, Gαi2 and Gαi3 10. Gαi proteins bind to GPCR and inhibit adenylate cyclase (AC), thereby depleting cyclic AMP (cAMP) 10. Such process can be blocked by pertussis toxin 11, 12. Pertussis toxin was shown to potently suppress HGG cell migration and proliferation 13. In addition, co-administration of pertussis toxin and temozolomide led to robust anti-glioma effect 14. Moreover, Gαi proteins activation increased malignant growth of glioma cells 12. These sporadic studies revealed a potential role of Gαi proteins in glioma growth and progression.

Interestingly, our group has shown that Gαi1 and Gαi3 are vital proteins mediating signaling transduction for receptor tyrosine kinases (RTKs), including VEGFR2 15, EGFR 16 and FGFR 17 as well as KGFR 18 and TrkB 19. Gαi1 or Gαi3 are recruited to ligand-activated RTKs, mediating downstream signaling cascades (Akt-mTORC1/Erk-MAPK) activation 15-19. We found that Gαi1/3-RTKs association was important for Akt-mTORC1 activation, and more importantly, glioma growth. Contrarily, Gαi1/3 silencing, Cas9-sgRNA knockout (KO) or mutation hindered Akt-mTOR activation and suppressed malignant behaviors of glioma cells 17, 20, 21. Moreover, Gαi1 and Gαi3 upregulation in human glioma correlates with patients' clinical parameters 17, 20, 21.

Intriguingly, depletion of Gαi2 was unable to prevent downstream signaling activation by RTKs 15-19. Yet a potential function of Gαi2 in carcinogenesis and tumor progression has been reported. Gαi2 is elevated in colitis-associated cancer (CAC), correlating with decreased relapse-free survival 22. Conversely, conditional knockdown of Gαi2 in CD11c^+^ cells reduced CAC carcinogenesis 22. Yin *et al.,* reported that Gαi2 is important for epithelial ovarian cancer cell growth 23. Conversely, microRNA-222-3p silenced Gαi2 to arrest epithelial ovarian cancer cell growth 23. Zhang* et al.,* proposed a pivotal role of Gαi2 in the development of nonalcoholic steatohepatitis 24. Gαi2 expression was upregulated in liver tissues of NASH patients 24. Importantly, hepatocytes specific Gαi2-deficient mice were resistant to the development of steatohepatitis 24. Here we will show that overexpressed Gαi2 is important for glioma cell growth possibly by promoting activation of NFκB (nuclear factor kappa B) cascade.

## Materials and methods

### Reagents

Polybrene, BAY-11-7082, mithramycin A, antibiotics, serum, puromycin and cell culturing medium were from Sigma-Aldrich (St. Louis, MO). Antibodies and fluorescence probes were reported early 17, 20, 21, 25.

### Bioinformatics studies

The RNA-seq data, including 166 GBM (glioblastoma multiforme), 523 LGG (low grade glioma) tissues and 1157 normal tissues, along with the clinical data, were provided from UCSC XENA (https://xenabrowser.net/datapages/). Normalized gene expression was measured as ​transcripts per million reads plus the log2-based transformation. The overall survival of GBM and LGG patients was assessed through Kaplan-Meier analysis using the “Survival” along with “SurvMiner” R packages. The accuracy evaluation of the prognostic of Gαi2 was carried out by ROC curves using the R packages “Survival ROC” and “time ROC”. TCGA LGGGBM cohorts were thereafter analyzed and *Gαi2*-associated differentially expressed gene (DEGs) were retrieved. KEGG analyses were carried out to explore the enrichment pathways. Chinese glioma functional genomic data were retrieved from the Chinese Glioma Genome Atlas (CGGA) 26. The RNA sequencing of Diffuse Gliomas was through the Illumina Hiseq 2000. Clinical data were also retrieved from the CGGA data portal. RNA sequencing data and Clinical data were analyzed using R. software. The "Survival" package, “SurvMiner” package and "ggpubr" package were used.

### Human tissues and cells

Human tissues were reported previously 17, 20, 21, 25 and were tested as reported 17, 21. The primary human glioma cells (“P1-P5”, derived from five different patients), the primary human astrocytes (“Astrocytes1/2”), glioma cell lines (A172/U87MG/U251MG/SHG-44) were reported early 17, 21, 27, 28.

### shRNA or gene overexpression

Verified Gαi2 shRNA sequence, Sp1 shRNA sequence, *Gαi2* cDNA or *Sp1* cDNA [NM_138473.3] was packaged into a GV369 construct (no GFP) (from Genechem). The constructs were each transfected to HEK-293T cells together with the lentivirus envelope constructs (Genechem). Thereafter, the viral particles were filtered, enriched and transfected (at MOI = 15) to the indicated glioma cells or astrocytes. Cells were maintained under polybrene-containing complete medium, and stable cells formed after puromycin treatment for 96h. Overexpression or silencing of targeted genes was verified. Alternatively, Gαi2 shRNA/shC (“the scramble control non-sense shRNA” 20, 25) was packed into the described adeno-associated virus (AAV) construct 20, 25, and shRNA AAV generated.

### Cas9-sgRNA (single guide RNA)-induced gene knockout

Cells were transfected with the pLV-hUbC-dCas9-VP64 lentiviral construct (GeneChem), and dCas9-expressing glioma cells were established after selection 29. Next, the verified sgRNA-CRISPR/dCas-9-Gαi2 lentiviral construct (Genechem) or the verified sgRNA-CRISPR/dCas-9-Sp1 lentiviral construct (Genechem) was transduced to dCas9-expressing cells, and stable cells formed following puromycin-mediated selection (for 96h). Gαi2/Sp1 KO was verified at mRNA and protein levels. The control cells were with the lenti-CRISPR/dCas-9 empty vector (“Cas9C”) 20, 25.

### Cellular functions and gene/protein detection

CCK-8, colony formation, “Transwell” cell migration and “Matrigel Transwell” invasion assays, were reported early 21, 30-34. The caspase activity assay, Histone DNA ELISA, nuclear TUNEL fluorescence staining and JC-1 monomer staining of mitochondrial depolarization were reported early 21, 30-35. mRNA detection by qRT-PCR and protein detection by Western blotting were reported previously 21, 30-34. Figure **S1** listed the uncropped blotting images.

### NFκB activity

Briefly, the nuclear proteins were extracted through high-speed centrifugation. The TransAM™ ELISA kit (Active Motif) was utilized to examine the NFκB (p65) DNA-binding activity. In brief, 0.25 μg of nuclear extracts were tested for the binding of p65 to the specific DNA sequence. Following colorimetric reaction, the optical density (OD) value was tested through ELISA at 450 nm.

### Chromatin immunoprecipitation (ChIP)

The detailed protocols of ChIP assay were reported in our previous study 25, 30. Briefly, the cell and human tissue lysates were homogenized 36 and fragmented DNA was achieved. Lysates were immunoprecipitated (IP) with the anti-Sp1 antibody. Sp1-bound DNA with the Gαi2 promoter site 37 was tested by the quantitative PCR method.

### Xenograft studies

The nude mice were reported previously 17, 21. P1 cells or A172 cells were subcutaneously injected into the right flanks of nude mice and glioma xenografts were formed. The mice were intratumorally injected with the Gαi2 shRNA-containing AAV or control shRNA AAV (1×10^9^ PFU). Alternatively, P1 glioma cells were intracranially injected to the brains of the nude mice as described 38 and intracranial P1 glioma xenografts were formed. The protocols of this study were approved by Soochow University's IACUC and Ethics Committee.

### Statistical analyses

Statistical methods were reported in our previous studies 20, 25. The data in this study were normally distributed and were shown as mean ± standard deviation (SD). All *in vitro* experiments were repeated five times, and each time similar results obtained.

## Results

### Gαi2 overexpression in human glioma

**Figure 1:**
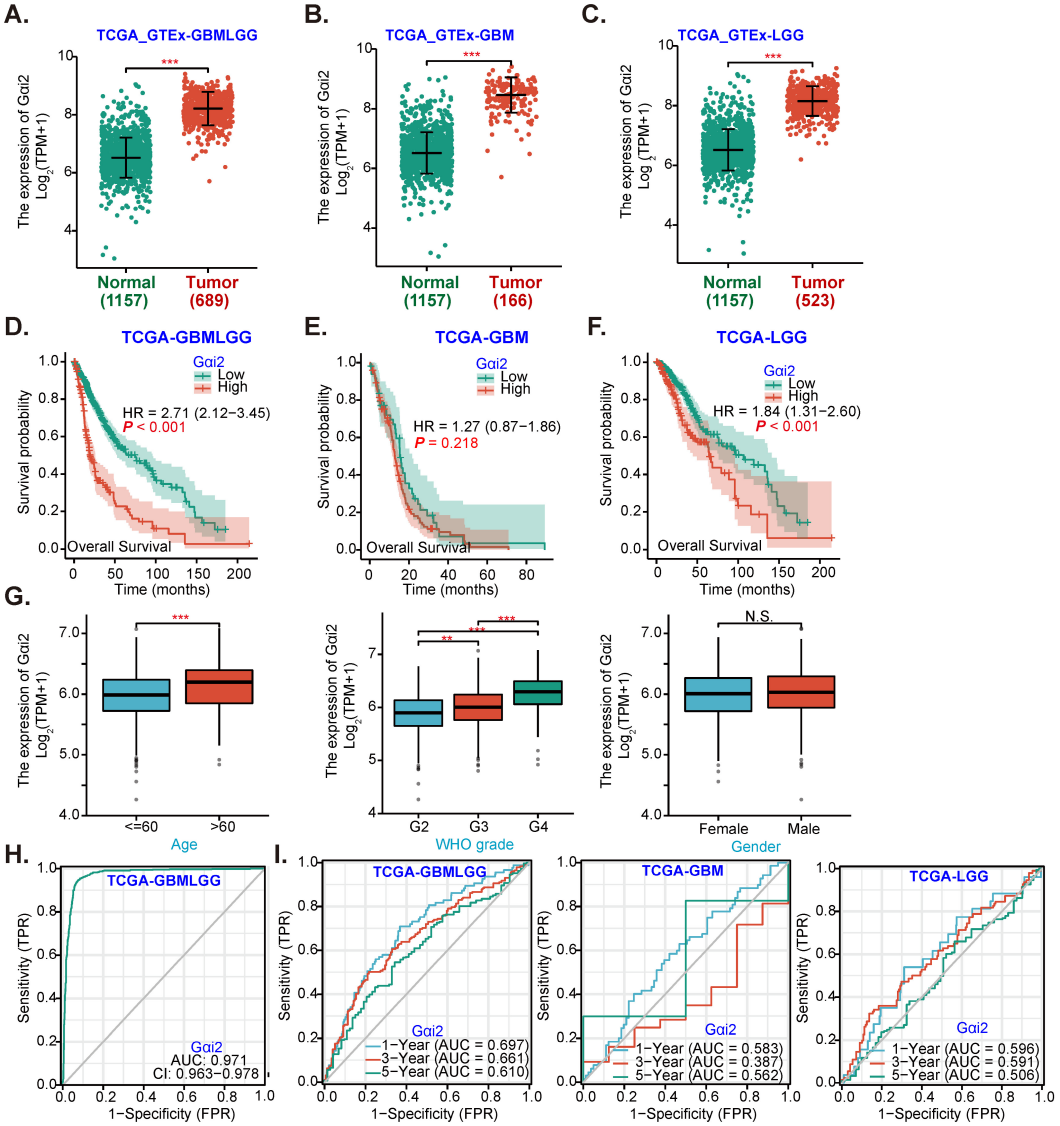
** Gαi2 overexpression in human glioma.** TCGA cohorts plus GTEx project revealed *Gαi2* transcripts in 689 glioma tissues (“Tumor”), including 166 glioblastoma (GBM) tissues and 523 low grade glioma (LGG) tissues as well as in 1157 normal brain tissues (“Normal”) (**A**-**C**). Kaplan Meier Survival analyses of TCGA cohorts based on Gαi2 expression in glioma (GBMLGG) patients (**D**), GBM patients (**E**) and LGG patients (**F**). The subgroup analyzing *Gαi2* expression and glioma patients' clinical characteristics in TCGA GBMLGG cohorts were shown (**G**). Nomogram for high glioma *Gαi2* expression in predicting overall survival probability of GBMLGG patients (**H**), and in predicating 1-/3-/5-year overall survival probability of GBMLGG/GBM/LGG patients was shown (**I**). “TPM” stands for transcripts per million. “AUC” stands for area under curve. ***P < 0.001; **P < 0.001; *P < 0.05; “N. S.” means ***P*** > 0.05.

Gαi2 expression data were retrieved from TCGA and Genotype-Tissue Expression (*GTEx*) project through UCSC XENA. A total of 689 glioma tissues (“Tumor”), including 166 glioblastoma (GBM) tissues and 523 low grade glioma (LGG) tissues, as well as 1157 normal brain tissues (“Normal”) were retrieved. *Gαi2* transcript number in 689 glioma tissues (GBMLGG, “Tumor”) was higher than it in the normal brain tissues (***P*** < 0.001, Figure **1A**). Further analyses revealed that *Gαi2* transcripts were significantly elevated in both GBM tissues (Figure **1B**) and LGG tissues (Figure **1C**).

The Kaplan-Meier survival and univariate Cox analysis from TCGA revealed that high *Gαi2* expression in glioma tissues (GBMLGG) was correlated with poor patients' overall survival (***P*** < 0.001, Figure **1D**). Compared to *Gαi2*-low GBM patients,* Gαi2*-high GBM patients tend to have poor overall survival (Figure **1E**), although no significant different was detected (***P*** = 0. 218, Figure **1E**). Importantly,* Gαi2*-high LGG patients' overall survival is significantly lower (Figure **1F**). Moreover, *Gαi2* overexpression in GBMLGG tissues is correlated with patients' age and higher tumor grade (Figure **1G**), but was not correlated with patients' gender (Figure **1G**).

Alignment Diagram (Nomogram) prediction map based on *Gαi2* expression showed that *Gαi2* overexpression had a significant value in predicting poor survival probability of patients with GBMLGG (Figure **1H**), with area under the survival curve (AUC) at: 0.971 (Figure **1H**). Moreover, *Gαi2* overexpression could predict poor 1/3/5-year survival probability of patients with GBMLGG, GBM or LGG (Figure **1I**). Bioinformatics analyses show that *Gαi2* is significantly elevated in human glioma, correlating with patients' poor survival and higher grade of the tumors.

### Gαi2 is overexpressed in local glioma tissues and cells

**Figure 2:**
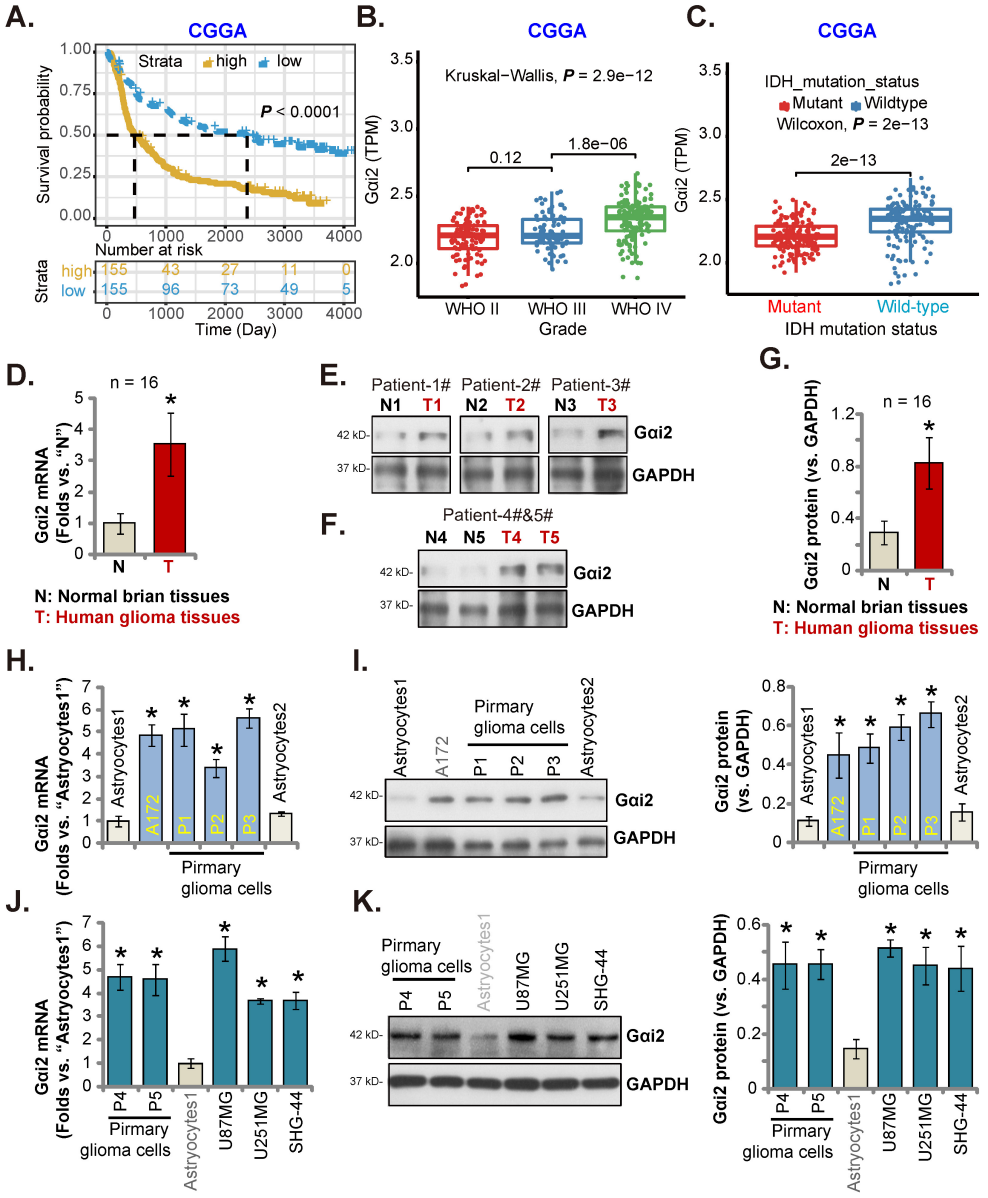
** Gαi2 is overexpressed in local glioma tissues and cells.** Kaplan Meier Survival analyses from Chinese Glioma Genome Atlas (CGGA) database based on Gαi2 expression in glioma patients were shown (**A**). CGGA cohorts showed that *Gαi2* mRNA overexpression in glioma patients correlated with high tumor grade (**B**) and WT IDH status (**C**). Gαi2 expression in glioma tissues (“T”) and adjacent normal brain tissues (“N”) of 16 local HGG patients was shown (**D**-**G**). *Gαi2* mRNA and protein expression in astrocytes (“Astrocytes1/2”), the immortalized (A172, U87MG, U251MG and SHG-44) and primary (“P1”, “P2”, “P3”, “P4” and “P5”) glioma cells was shown (**H**-**K**). *P < 0.05 versus “N” tissues/“Astrocytes1”.

CGGA database revealed that high-*Gαi2* expression glioma patients have lower survival (***P*** < 0.001, Figure **2A**). Moreover, in HGG tissues* Gαi2* expression was significantly higher than that in low grade (grade II) glioma tissues (Figure **2B**). Importantly, in glioma tissues* Gαi2* overexpression correlated with wild-type (WT) IDH status (***P*** < 0.001, Figure **2C**), and low *Gαi2* expression was detected in IDH mutant glioma tissues (Figure **2C**).

In local human glioma tissues Gαi2 expression was tested. Sixteen (n = 16) HGG tissues (labeled as “T”) and matched adjacent normal brain tissues (labeled as “N”) 17, 20, 21 were tested. Figure **2D** revealed that *Gαi2* mRNA levels in glioma tissues were increased significantly. In five representative HGG patients (Patient-1#-5#), Gαi2 protein expression was elevated in the glioma tissues (Figure **2E** and **F**). Combining all 16 pairs of Gαi2 expression data showed that Gαi2 protein was significantly upregulated in glioma tissues (Figure **2G**).

We next studied whether Gαi2 was upregulated in different human glioma cells, including primary human glioma cells (“P1-P5”, derived from five patients 21) and A172 cells. *Gαi2* mRNA levels in the tested glioma cells were significantly higher than those in the primary human astrocytes (“Astrocytes1/2”) (Figure **2H**). *Gαi2* protein upregulation was also in primary and immortalized glioma cells (Figure **2I**). *Gαi2* mRNA (Figure **2J**) and protein (Figure **2K**) expression was also significantly elevated in other primary glioma cells-derived from other patients (“P4”/“P5”) and in immortalized cell lines, including U87MG, U251MG and SHG-44. These results clearly show that Gαi2 is overexpressed in local glioma tissues/cells.

### Gαi2 depletion leads to robust anti-glioma cell activity

**Figure 3:**
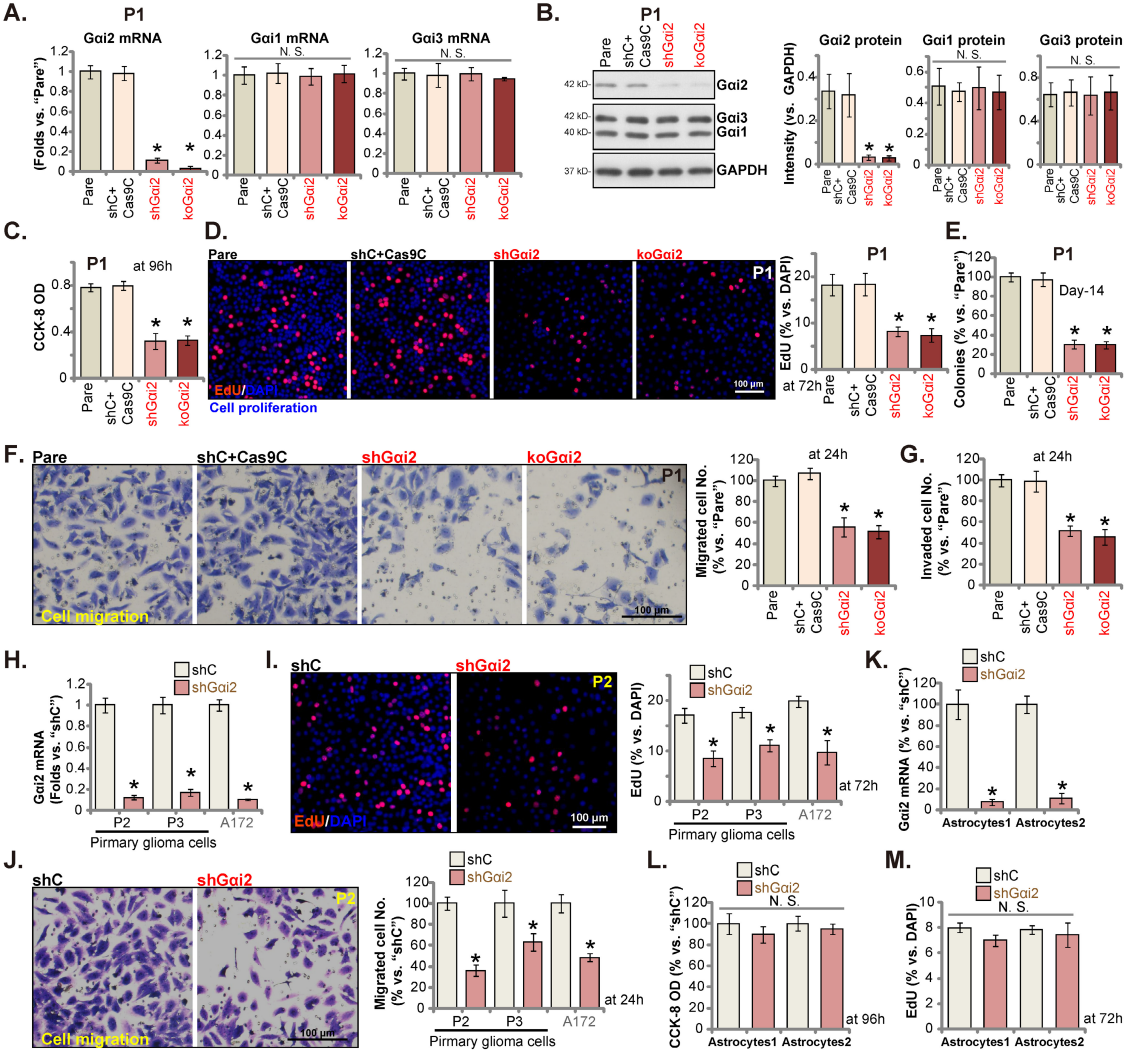
** Gαi2 depletion leads to robust anti-glioma cell activity.** Puromycin-selected stable P1 glioma cells, with the lentiviral Gαi2 shRNA (“shGαi2”), the CRISPR/dCas9-Gαi2-KO construct (“koGαi2”) or the scramble non-sense control shRNA (“shC”) plus the CRISPR/dCas9 empty construct (“Cas9C”), were formed, and expression of listed mRNAs and proteins was tested (**A** and **B**). After culturing for designated time periods, cell viability (**C**), nuclear EdU incorporation (**D**) and colony formation (**E**), as well as cell *in vitro* cell migration (**F**) and invasion (**G**) were tested, and results quantified. Puromycin-selected stable P2/P3 primary glioma cells, A172 glioma cells (**H**-**J**), or primary human astrocytes (“Astryocyte1” and “Astryocyte2”, **K**-**M**), with shGαi2 or shC, were formed, *Gαi2* mRNA expression was shown (**H** and **K**). After culturing for designated time periods, cellular functions including EdU incorporation (**I** and **M**), *in vitro* cell migration (**J**) and cell viability (**L**) were tested. “Pare” are parental control cells. * ***P*** < 0.05 *vs.* “Pare” cells /“shC” treatment. “N.S.” means ***P*** > 0.05. Scale bar = 100 μm.

We next tested Gαi2's potential functions. P1 primary human glioma cells, as reported previously 17, 20, 21, 25, were infected with Gαi2 shRNA-expressing lentiviral particles. Stable P1 glioma cells were thereafter formed after selection, namely“shGαi2” cells. Alternatively, the lentiviral particles with the CRISPR/dCas9-Gαi2-KO construct were added to P1 glioma cells with dCas9, and stable cells formed and named “koGαi2” cells. The control P1 glioma cells were with the scramble control shRNA (non-sense) plus the CRISPR/dCas9 empty construct (“shC+Cas9C”). As shown, *Gαi2* mRNA and protein (Figure **3A** and **B**) levels were significantly decreased in shGαi2 and koGαi2 P1 glioma cells, and Gαi1/3 expression unchanged (Figure **3A** and **B**). CCK-8 viability was decreased significantly in shGαi2 and koGαi2 P1 glioma cells (Figure **3C**). Gαi2 shRNA or KO largely inhibited P1 glioma cell proliferation and significantly decreased the EdU-stained nuclei (Figure **3D**). In addition, genetic depletion of Gαi2 prevented P1 glioma cell colony formation (Figure **3E**), further supporting the anti-proliferative activity.

Next, the *in vitro* cell migration and the *in vitro* cell invasion of P1 glioma cells were tested separately by “Transwell” (Figure **3F**) and “Matrigel Transwell” (Figure **3G**) assays. Following Gαi2 depletion, the mobility of P1 glioma cells was largely inhibited (Figure **3F** and **G**). Notably, the “shC+Cas9C” treatment failed to significantly change Gαi1/2/3 expression (Figure **3A** and **B**) as well as P1 glioma cell functions (Figure **3C**-**G**).

Other primary human glioma cells, “P2” and “P3” (see our previous studies 17, 20, 21, 25), as well as the immortalized A172 glioma cells were again infected Gαi2 shRNA-expressing lentiviral particles, and stable cells (labeled as “shGαi2”) were established after selection, showing depleted *Gαi2* mRNA (Figure **3H**). Gαi2 shRNA significantly inhibited cell proliferation and decreased EdU incorporation in these primary and immortalized glioma cells (Figure **3I**). Moreover, *in vitro* cell migration was significantly slowed following Gαi2 silencing (Figure **3J**). These results clearly supported that Gαi2 depletion led to robust anti-glioma cell activity.

Gαi2 shRNA lentiviral particles were also added to the primary human astrocytes (“Astryocyte1” and “Astryocyte2”) 17, 20, 21, and stable “shGαi2” astrocytes were formed after puromycin selection. Robust *Gαi2* mRNA silencing was detected in shGαi2 astrocytes (Figure **3K**). Gαi2 shRNA, however, did not alter CCK-8 viability (Figure **3L**) and proliferation/EdU incorporation (Figure **3M**) in the primary astrocytes.

### Gαi2 depletion provokes apoptosis in glioma cells

**Figure 4:**
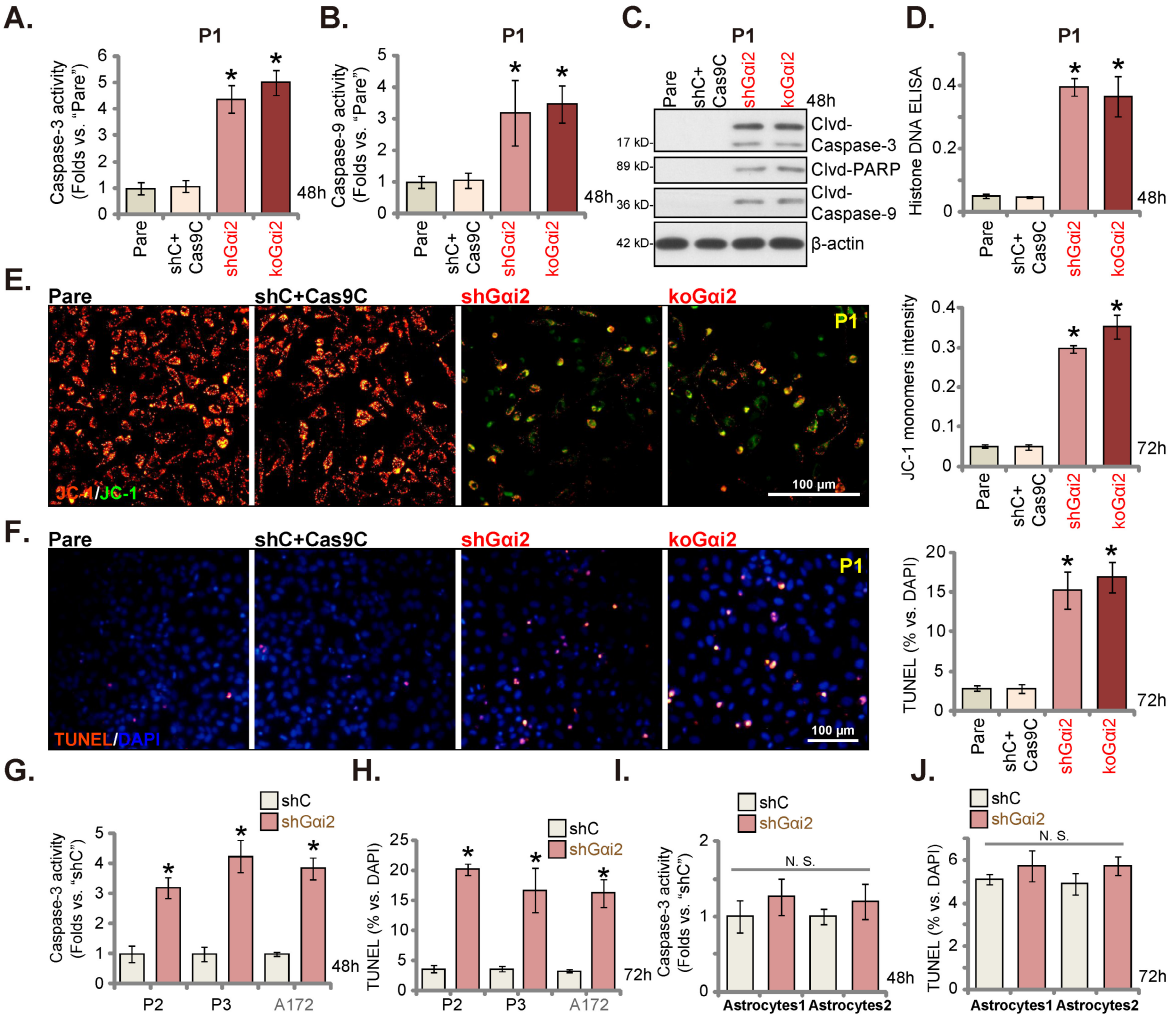
** Gαi2 depletion provokes apoptosis in glioma cells.** Puromycin-selected stable P1 glioma cells, with the lentiviral Gαi2 shRNA (“shGαi2”), the CRISPR/dCas9-Gαi2-KO construct (“koGαi2”) or the scramble non-sense control shRNA (“shC”) plus the CRISPR/dCas9 empty construct (“Cas9C”), were formed. After culturing for designated time periods, the caspase-3/-9 activities (**A** and **B**), apoptosis-associated proteins (**C**) and Histone DNA contents (**D**) were measured, with JC-1 green monomers measured (**E**); Cell apoptosis was examined by nuclear TUNEL staining (**F**). Puromycin-selected stable P2/ P3 primary glioma cells, A172 glioma cells (**G** and **H**), or primary human astrocytes (“Astryocyte1” and “Astryocyte2”, **I** and **J**), with shGαi2 or shC, were formed and cultured, the caspase-3 activity (**G** and **I**) and cell apoptosis (**H** and **J**) were similarly tested. “Pare” are parental control cells. * ***P*** < 0.05 *vs.* “Pare” cells /“shC” treatment. “N.S.” means ***P*** > 0.05. Scale bar = 100 μm.

Gαi2 depletion led to robust anti-glioma cell activity, causing viability reduction, proliferation inhibition, G1-S arrest and mobility reduction. Its role on cell apoptosis was tested as well. In both shGαi2 and koGαi2 P1 glioma cells (see Figure **3**), the activity of caspase-3/-9 was augmented significantly (Figure **4A** and **B**). Caspase-3, caspase-9 and poly (ADP-ribose) polymerase-1 (PARP) cleavages were induced in Gαi2-silenced/KO P1 glioma cells (Figure **4C**), and levels of histone-bound DNA were augmented (Figure **4D**). Gαi2 shRNA/KO induced mitochondrial depolarization in P1 cells, causing the transition of JC-1 red fluorescence to green fluorescence (monomers) (Figure **4E**). Significantly, Gαi2 depletion provoked apoptosis in P1 cells and the TUNEL stained nuclei were increased in shGαi2 and koGαi2 cells (Figure **4F**). The shC+Cas9C control treatment, as expected, failed to induce caspase activation (Figure **4A**-**D**), mitochondrial depolarization (Figure **4E**) and apoptosis (Figure **4F**) in P1 glioma cells.

Similar results were obtained in P2/P3 primary human glioma cells and A172 cells. As shown shRNA-induced silencing of Gαi2 (“shGαi2”, see Figure **3**) increased caspase-3 activity (Figure **4G**) and the TUNEL nuclei number (Figure **4H**). Whereas in the “Astryocyte1” and “Astryocyte2”, Gαi2 shRNA (“shGαi2”, see Figure **3**) failed to boost the caspase-3 activity (Figure **4I**) and nuclear TUNEL staining ratio (Figure **4J**).

### Ectopic Gαi2 overexpression promotes glioma cell growth

**Figure 5:**
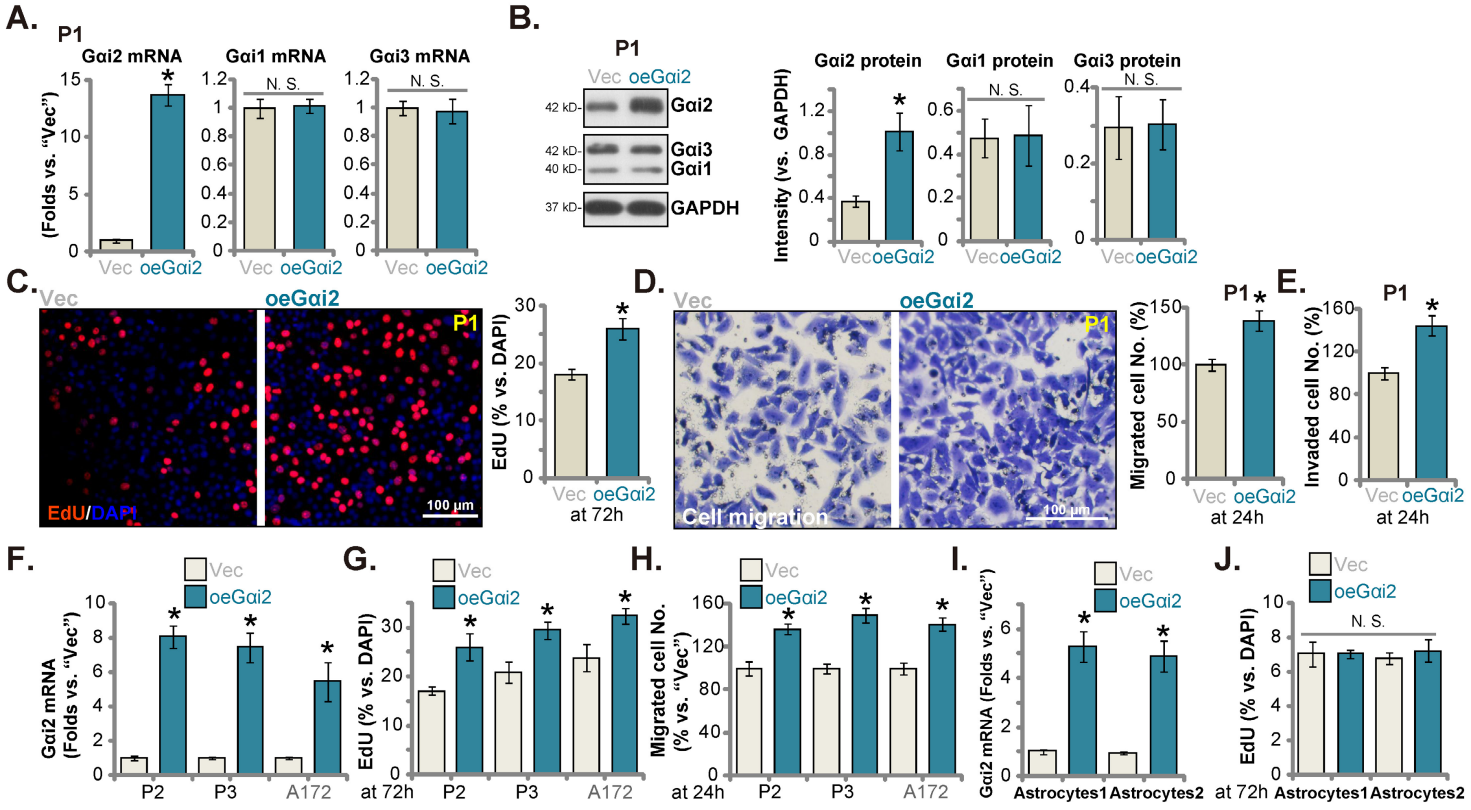
** Ectopic Gαi2 overexpression promotes glioma cell growth.** Puromycin-selected stable P1 glioma cells, with the lentiviral Gαi2-expressing construct (“oeGαi2”) or the vector (“Vec”), were formed, and listed mRNAs and proteins were shown (**A** and **B**). After culturing for indicated time periods, cellular functions, including nuclear EdU incorporation (**C**), *in vitro* cell migration (**D**) and invasion (**E**) were tested. Puromycin-selected stable P2/P3 primary glioma cells, A172 glioma cells (**F**-**H**), or primary human astrocytes (“Astryocyte1” and “Astryocyte2”, **I** and **J**), with oeGαi2 or Vec, were formed and *Gαi2* mRNA expression was tested (**F** and **I**); After culturing, EdU incorporation (**G** and **J**) and *in vitro* cell migration (**H**) were tested similarly, with results quantified. * ***P*** < 0.05 *vs.* “Vec” cells. “N.S.” means ***P*** > 0.05. Scale bar = 100 μm.

Ectopic Gαi2 overexpression could possibly further enhance glioma cell progression. Therefore, the lentiviral particles packaging the Gαi2-expressing construct were transfected to P1 cells. Stable cells were again formed after selection: “oeGαi2”. *Gαi2* mRNA level was significantly augmented in oeGαi2 cells and was over 13-fold higher than that of vector control P1 glioma cells (“Vec”) (Figure **5A**). Figure **5B** confirmed Gαi2 protein upregulation in oeGαi2 P1 glioma cells. Gαi1/3 expression was not changed (Figure **5A**-**B**). oeGαi2 promoted P1 glioma cell proliferation and increased nuclear EdU incorporation (Figure **5C**). Cell *in vitro* migration and invasion (Figure **5D**-**E**) were accelerated following ectopic Gαi2 overexpression. The Gαi2-expressing construct lentiviral particles were also added to P2/P3 primary cells and A172 cells, resulting in robust *Gαi2* mRNA elevation (“oeGαi2”) (Figure **5F**). In the tested primary and immortalized glioma cells, ectopic Gαi2 overexpression augmented cell proliferation (EdU incorporation, Figure **5G**) and accelerated *in vitro* cell migration (Figure **5H**). Thus, Gαi2 overexpression resulted in pro-glioma cell activity. Whereas in the “Astryocyte1” and “Astryocyte2”, ectopic Gαi2 overexpression, by the same lentiviral Gαi2-expressing construct, led to Gαi2 *mRNA* upregulation (“oeGαi2”, Figure **5I**). It however failed to increase proliferation (EdU incorporation, Figure **5J**) in the astrocytes.

### Gαi2 is important for NFκB activation in glioma cells

**Figure 6:**
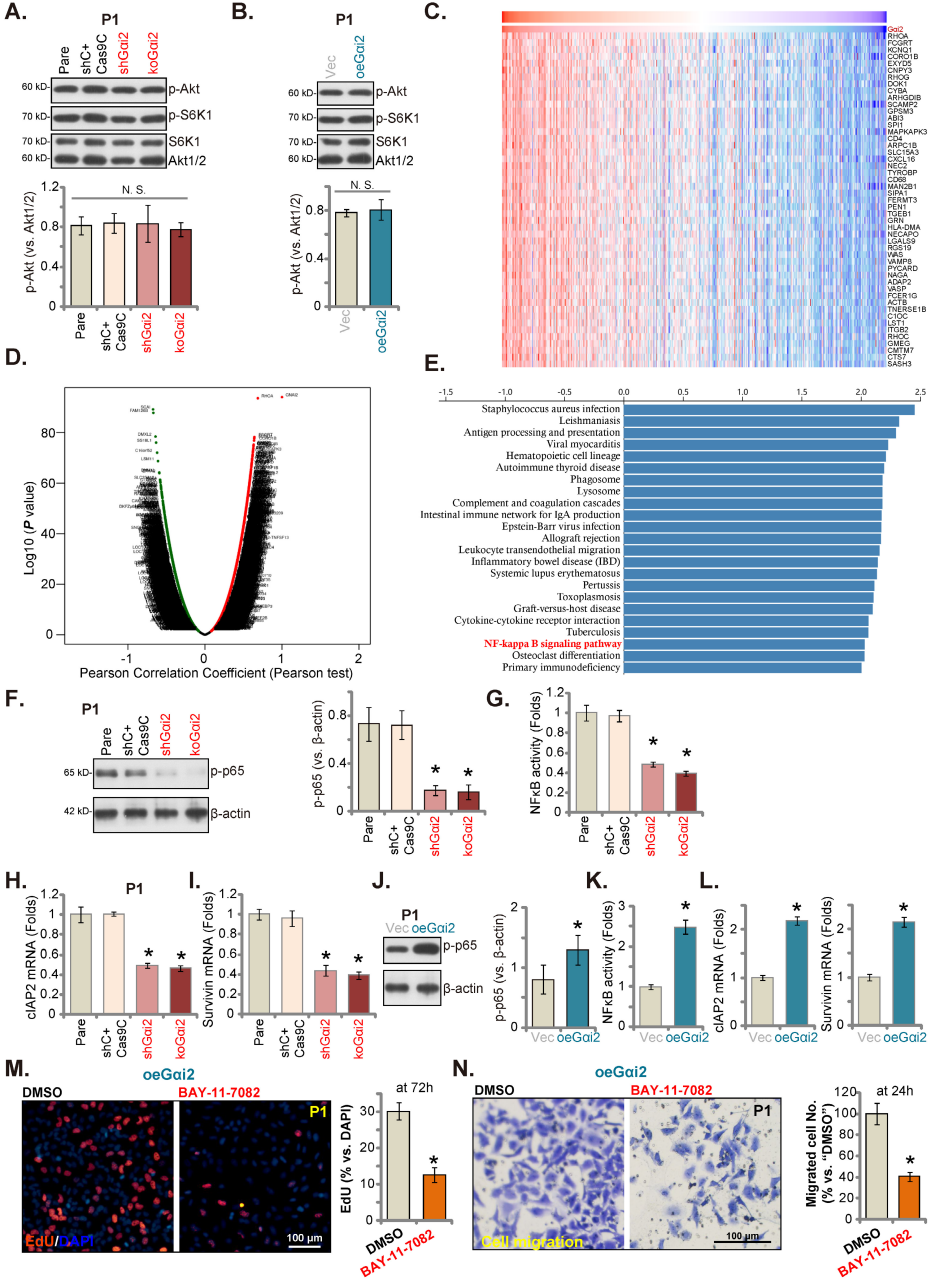
** Gαi2 is important for NFκB activation in glioma cells.** Expression of listed mRNAs/proteins in the stable P1 glioma cells with designated Gαi2 genetic modifications was tested (**A**, **B**, **F**, **H**-**J** and **L**); The p65 DNA-binding activity was tested as well (**G** and **K**). Differentially expressed gene (DEGs) based on *Gαi2* expression in TCGA LGGGBM cohorts were shown (**C**) and the volcano map of DEGs was presented (**D**); KEGG analyses of *Gαi2*-associated DEGs and the corresponding enriched pathways were listed (**E**). The oeGαi2 P1 glioma cells were treated with BAY-11-7082 (15 μM) and cultured for designated hours, cell proliferation (nuclear EdU staining, **M**) and *in vitro* cell migration (**N**) were measured. *P < 0.05 versus “Pare” cells/“Vec” cells or “DMSO” treatment. Scale bar = 100 μm.

Gαi1/3 association with multiple RTKs (and non-RTK receptors) mediates Akt-mTOR cascade activation 15, 16, 18, 19, 21, 31, 39. In P1 glioma cells, Gαi2 shRNA or KO however failed to significantly inhibit phosphorylation of Akt and S6K1 (Figure **6A**). Moreover, Akt-S6K1 phosphorylation was unchanged following ectopic Gαi2 overexpression (Figure **6B**). TCGA LGGGBM cohorts were thereafter analyzed and *Gαi2*-associated differentially expressed gene (DEGs) were retrieved (Figure **6C**). The volcano map of *Gαi2*-assocaited DEGs was presented in Figure **6D** (|LogFC|>1, Adjust ***P***-value < 0.05). KEGG enrichment pathway analyses found that *Gαi2*-associated DEGs were enriched in multiple signaling cascades (Figure **6E**). One key cascade is NFκB, important for glioma tumorigenesis and progression 40-42.

We thus tested NFκB activation in glioma cells. As shown, in both shGαi2 and koGαi2 P1 glioma cells (see Figure **3** and **4**), phosphorylated p65 levels were robustly decreased (Figure **6F**), the NFκB (p65) DNA-binding activity was also reduced in Gαi2-depleted P1 glioma cells (Figure **6G**). Moreover, NFκB-dependent oncogenic genes, including *cIAP2* and *survivin* 43-47, were downregulated in shGαi2 and koGαi2 P1 glioma cells (Figure **6H** and **I**). Conversely, phosphorylated p65 (Figure **6J**), NFκB (p65) DNA-binding activity (Figure **6K**) and *cIAP2*-*survivin* mRNA levels (Figure **6L**) were significantly augmented in oeGαi2 P1 glioma cells. Thus, Gαi2 is important for NFκB activation in glioma cells. BAY-11-7082, a well-known NFκB blocker 48, 49, substantially suppressed proliferation (Figure **6M**) and migration (Figure **6N**) of oeGαi2 P1 glioma cells. Thus, Gαi2-driven glioma cell progression is mediated, at least in part, through promoting NFκB cascade activation.

### Sp1 and *Gαi2* promoter binding increases in glioma tissues and cells

**Figure 7:**
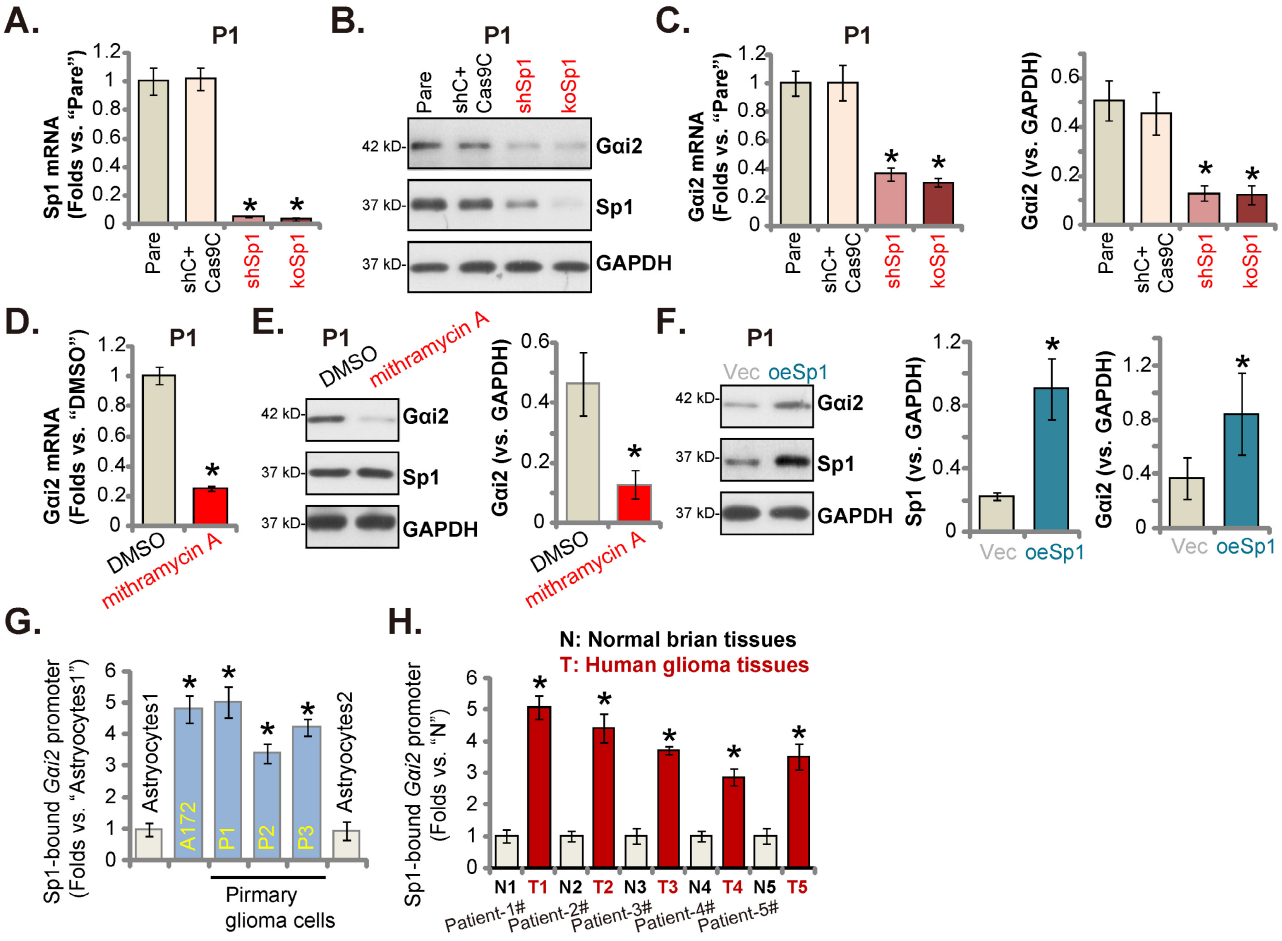
** Sp1 and *Gαi2* promoter binding increases in glioma tissues and cells.** Puromycin-selected stable P1 glioma cells, with the lentiviral Sp1 shRNA (“shSp1”), the CRISPR/dCas9-Sp1-KO construct (“koSp1”) or “shC plus Cas9C”, the lentiviral Sp1-expressing construct (“oeSp1”) or “Vec” were formed, and listed mRNAs and proteins were tested (**A**-**C** and **F**). P1 primary glioma cells were treated with mithramycin A (200 nM) or DMSO (0.1%) for 24h, and listed mRNAs and proteins were shown (**D** and **E**). Chromosome IP (ChIP) showed the relative amount of *Gαi2* promoter DNA binding to the transcription factor Sp1 in the listed cells (**G**) and human tissues (**H**). *P < 0.05 versus “Pare”/“Vec”/“DMSO”/“Astrocytes”/“N” tissues.

Since *Gαi2* mRNA/protein levels were both elevated in glioma, it could be due to the transcriptional mechanism. Recent studies have implied that Sp1 (specificity protein 1) could be an important transcription factor of Gαi2 37, 50. We first tested whether Sp1 was important for Gαi2 expression in glioma cells. To this purpose, lentiviral particles with Sp1 shRNA were added to P1 cells, and stable cells (“shSp1”) formed. Alternatively, the lentiviral particles with the CRISPR/dCas9-Sp1-KO construct was added to the dCas9-expressing P1 cells, and stable Sp1 KO cells (“koSp1”) formed after selection. *Sp1* mRNA (Figure **7A**) and protein (Figure **7B**) expression was robustly decreased in shSp1 and koSp1 P1 glioma cells. Importantly, *Gαi2* mRNA/protein (Figure **7B** and **C**) levels were reduced in Sp1-depleted P1 glioma cells. Mithramycin A, a compound that can prevent Sp1 binding to GC boxes in DNA 50, also decreased *Gαi2* mRNA/protein levels in P1 cells (Figure **7D** and **E**).

Next, the lentiviral particles packaging Sp1-overexpressing construct were added to P1 glioma cells, and stable cells established (“oeSp1”). Sp1 protein levels were remarkably upregulated in oeSp1 P1 glioma cells (Figure **7F**). Following Sp1 overexpression, Gαi2 protein expression was increased as well (Figure **7F**). Remarkably, Sp1 ChIP results revealed that Sp1-*Gαi2* promoter binding 51 in various glioma cells (“P1-P3” primary cells and A172 cells) was substantially higher than it in Astrocytes1/2 (Figure **7G**). Moreover, in human glioma tissues of five representative GBM patients, Sp1 binding to the *Gαi2* promoter was robustly increased (Figure **7H**). Therefore, Sp1 and *Gαi2* promoter binding increasing could be an important mechanism of Gαi2 upregulation in human glioma tissues and cells.

### Gαi2 silencing inhibits subcutaneous glioma xenograft growth in nude mice

**Figure 8:**
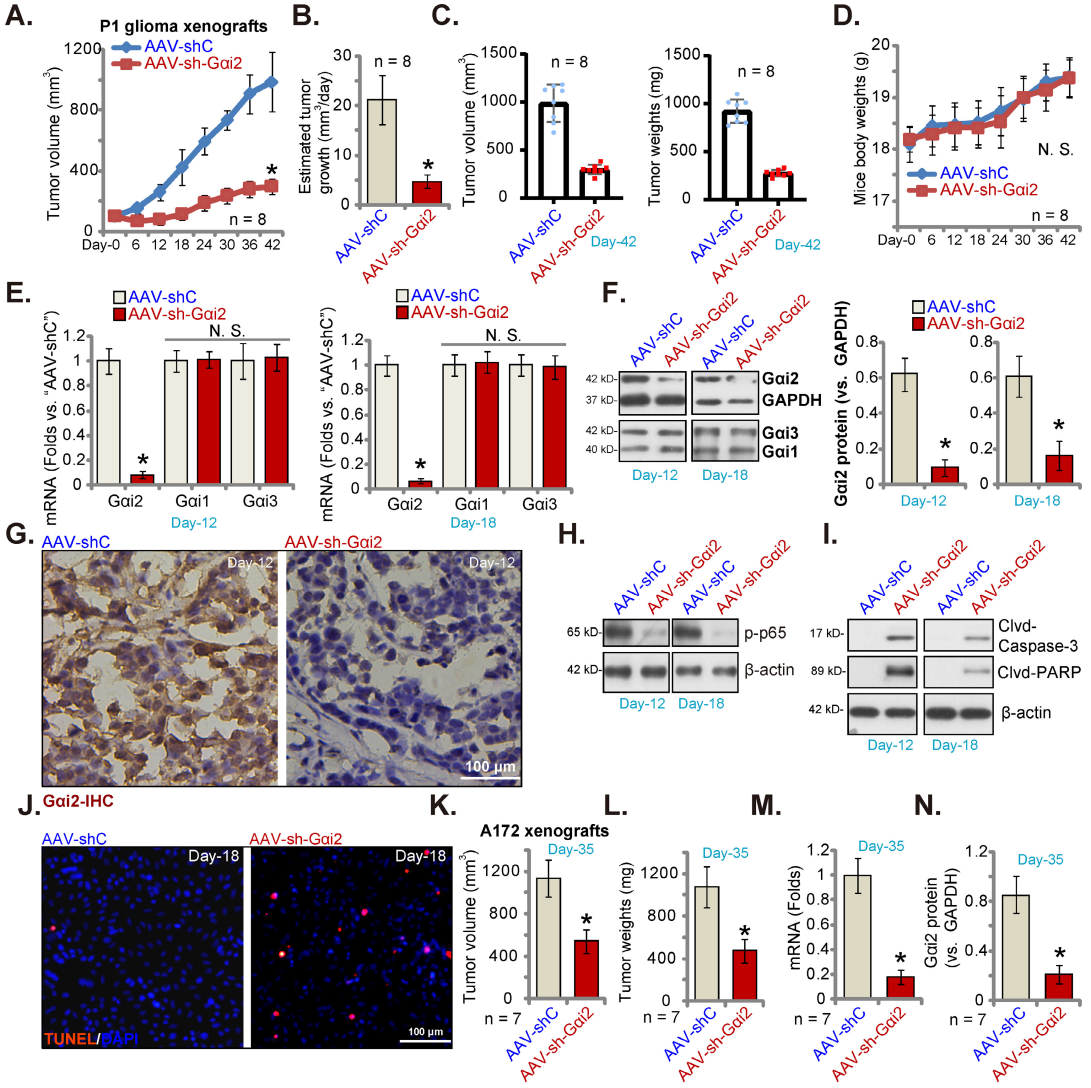
** Gαi2 silencing inhibits subcutaneous glioma xenograft growth in nude mice.** The subcutaneous P1 glioma xenograft-bearing nude mice were daily intratumorally injected with Gαi2 shRNA-expressing AAV (“AAV-sh-Gαi2”) or AAV-shC. The volumes of the xenografts (**A**) and animal body weights (**D**) were recorded. The estimated daily growth was calculated and was expressed at mm^3^ per day (**B**). At Day-42, all P1 glioma xenografts were isolated and measured (**C**). Listed mRNAs and proteins in the described P1 glioma xenograft tissues were tested (**E**, **F**, **H** and **I**). The representative IHC images of Gαi2 in the described P1 glioma xenograft slides were presented (**G**). Nuclear TUNEL fluorescence staining in the described P1 xenograft slides were presented (**J**). The subcutaneous A172 glioma xenograft-bearing nude mice were subject to daily intratumoral injection of AAV-sh-Gαi2 or AAV-shC. At Day-35, all A172 glioma xenografts were isolated, and were measured (**K** and **L**). In the xenograft tissues listed mRNAs and proteins were examined, and results quantified (**M** and **N**). *P < 0.05 versus “AAV-shC” group. “N.S.” means ***P*** > 0.05. Scale bar = 100 μm.

P1 glioma cells (five million cells per mouse) were subcutaneously (*s.c.*) injected to nude mice. Twenty days after cell injection, the subcutaneous P1 glioma xenografts were formed and each xenograft was close to 100 mm^3^ (“Day-0”). AAV with Gαi2 shRNA (“AAV-sh-Gαi2”) were intratumorally injected to P1 glioma xenografts daily (for ten days), and control mice intratumorally injected with AAV-shC. Every six days tumor volumes were recorded. As shown, AAV-sh-Gαi2 injection remarkably hindered subcutaneous P1 glioma xenograft growth (Figure **8A**) and reduced the estimated daily tumor growth 33, 52. Intratumoral AAV-sh-Gαi2 injection slowed P1 glioma xenograft growth (Figure **8B**). All P1 xenografts were carefully isolated at Day-42 and were tested. AAV-sh-Gαi2 xenografts were much smaller and lighter than AAV-shC xenografts (Figure **8C**). No significant difference was observed in the mice body weights (Figure **8D**). Thus, Gαi2 silencing inhibited subcutaneous P1 glioma xenograft growth in nude mice.

On “Day-12” and “Day-18” of the animal experiment, we separated one P1 glioma xenograft from each group. *Gαi2* mRNA/protein (Figure **8E** and **F**) levels were robustly decreased in AAV-sh-Gαi2-injected xenograft tissues. Contrarily, Gαi1 and Gαi3 expression was not changed (Figure **8E** and **F**). IHC studies confirmed Gαi2 protein silencing in AAV-sh-Gαi2 xenograft slides (at “Day-12”, Figure **8G**). Levels of phosphorylated p65, the indicator of NFκB activation, was significantly decreased in P1 glioma xenografts with AAV-sh-Gαi2 injection (Figure **8H**), whereas caspase-3 and PARP cleavages were augmented (Figure **8I**). Tissue immunofluorescence staining showed that TUNEL-stained nuclei were significantly increased in AAV-sh-Gαi2-injected P1 glioma xenografts (at “Day-18”, Figure **8J**), supporting apoptosis activation.

Next, A172 glioma cells (six million cells in each mouse) were *s.c.* injected to the nude mice, and A172 glioma xenografts formed after two weeks (100 mm^3^ at “Day-0”). The A172 xenograft-bearing nude mice were then subject to the same AAV-sh-Gαi2 injection or AAV-shC injection (daily for 10 days). After five weeks (“Day-35”), all A172 xenografts were isolated. As demonstrated, A172 xenografts with AAV-sh-Gαi2 injection were significantly smaller (Figure **8K**) and lighter (Figure **8L**) than AAV-shC-injected xenografts. Analyzing A172 xenograft tissues confirmed *Gαi2* mRNA (Figure **8M**) and protein (Figure **8N**) silencing in the AAV-sh-Gαi2 A172 xenografts.

### Gαi2 knockout hinders intracranial glioma xenograft growth in nude mice

**Figure 9:**
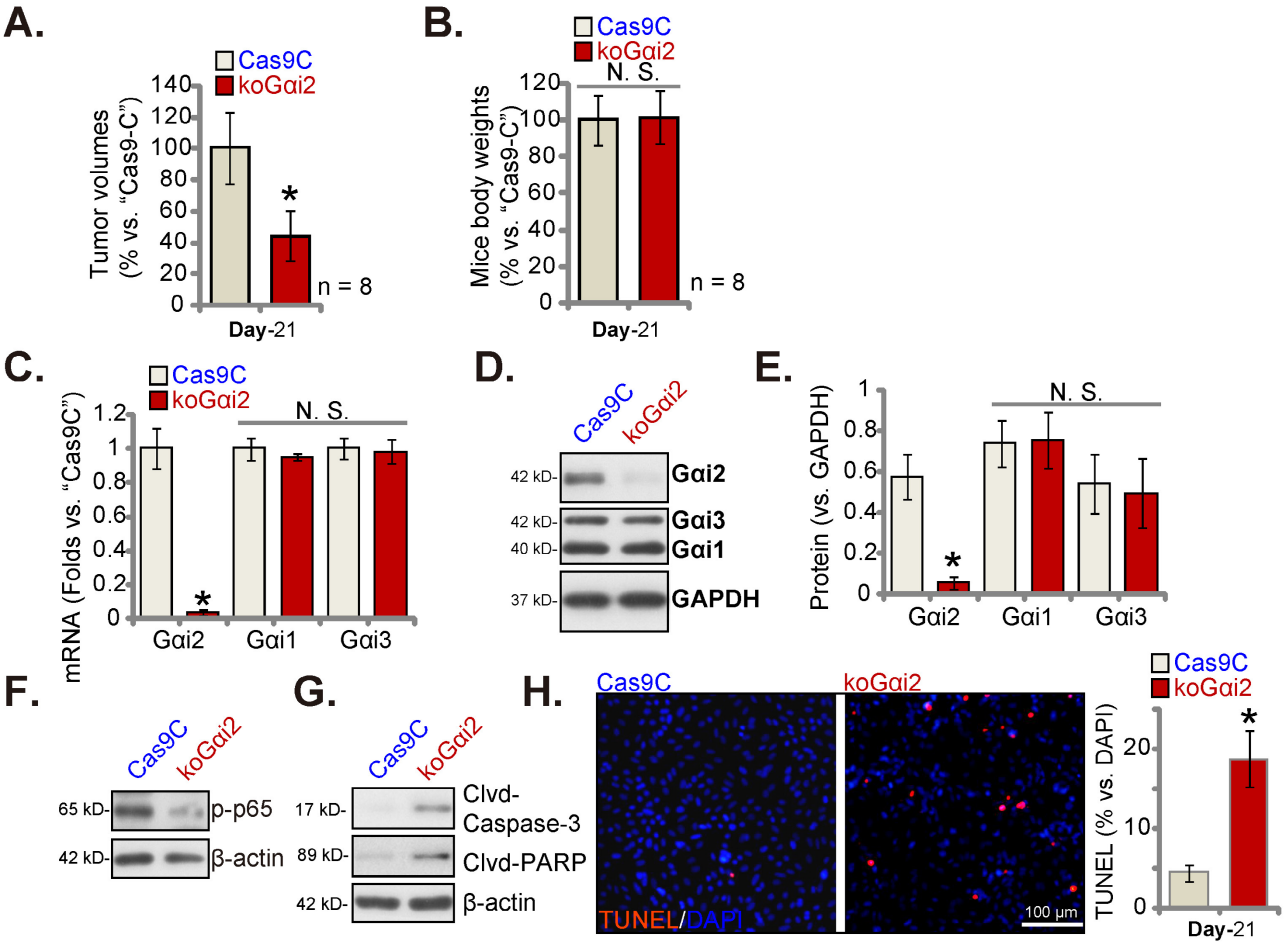
** Gαi2 knockout hinders intracranial glioma xenograft growth in nude mice.** P1 primary human glioma cells, at half million cells of each mouse, with the CRISPR/dCas9-Gαi2-KO construct (“koGαi2”) or control (“Cas9C”), were intracranially injected to nude mice's brains; After 21 days (“Day-21”), animals were decapitated and intracranial glioma xenografts were isolated, the tumor volumes (**A**) and mice body weights (**B**) were shown. The listed mRNAs and proteins were measured (**C**-**G**); Nuclear TUNEL fluorescence staining in the described intracranial P1 glioma xenograft slides were presented (**H**). *P < 0.05 versus “Cas9C” group. “N.S.” means ***P*** > 0.05. Scale bar = 100 μm.

Lastly, using the described protocol 17 CRISPR/dCas9-Gαi2-KO construct (“koGαi2”)-expressing P1 glioma cells or Cas9C control cells were injected intracranially into the brains of the nude mice. Five days later, the intracranial glioma xenograft was established 17, 20, 25. After 21 days (“Day-21”) the first Cas9C group mouse showed apparent symptoms. All mice were sacrificed and intracranial xenografts were isolated 17. The koGαi2 intracranial glioma xenografts were smaller than Cas9C intracranial xenografts (Figure **9A**). Mice body weights were indifferent (Figure **9B**).

*Gαi2* mRNA/protein expression was significantly decreased in koGαi2 intracranial P1 glioma xenograft tissues (Figure **9C**-**E**). Gαi1/3 expression was again unchanged (Figure **9C**-**E**)*.* In koGαi2 xenografts, phosphorylated p65 was decreased, indicating NFκB inactivation (Figure **9F**). Whereas caspase-3-PARP cleavages were significantly increased (Figure **9G**). Moreover, TUNEL-stained nuclei were robustly increased in koGαi2 intracranial P1 glioma xenografts (Figure **9H**), supporting apoptosis activation. These results showed that Gαi2 knockout hindered intracranial P1 glioma xenograft growth in nude mice.

## Discussion

GBM and other HGG are most aggressive and lethal malignant tumors that originate in the brain 53, 54. Currently, there is a lack of effective treatments 5, 6, 55, 56. Compared with traditional treatment methods, molecular targeted therapies 57, 58 could have better selectivity and specificity against glioma 5, 6, 55, 56. We showed that Gαi2 could be an important therapeutic oncotarget of glioma. Bioinformatics analyses revealed that *Gαi2* transcripts are significantly elevated in human glioma, and its overexpression correlates with poor patients' survival, higher tumor grade and WT-IDH status. Moreover, Gαi2 upregulation is also detected in local glioma tissues and various human glioma cells.

In primary and immortalized (A172) glioma cells, Gαi2 shRNA or KO substantially suppressed viability, cell proliferation and mobility. Silence of Gαi2 by targeted shRNA however failed to inhibit viability and proliferation in non-cancerous human astrocytes. In addition, Gαi2 shRNA or KO provoked caspase activation, mitochondrial depolarization and apoptosis in the primary and A172 glioma cells. Whereas Gαi2 silencing failed to provoke caspase-apoptosis activation in human astrocytes. Contrarily, ectopic Gαi2 overexpression, using the lentiviral construct, further promoted malignant behaviors of primary and immortalized glioma cells, enhancing cell proliferation, migration and invasion. Gαi2 overexpression was however not effective in human astrocytes. Importantly, daily intratumoral Gαi2 shRNA AAV injection largely hindered subcutaneous P1 xenograft growth in nude mice. Moreover, the growth of intracranial P1 xenografts was largely inhibited after Gαi2 KO. Therefore, overexpressed Gαi2 is important for glioma cell growth.

Activation of NFκB cascade is important for carcinogenesis and progression of human glioma 40-42. Xu *et al.,* have shown that cullin-7 (CUL7), a DOC domain-containing cullin family protein, promoted gliomagenesis by promoting MST1 protein degradation and activating NF-κB pathway 42. Conversely, CUL7 silencing inhibited NF-κB activation and prevented growth of glioma cells 42. Chai *et al.,* reported that overexpressed YTHDF2 promoted glioma cell growth by activating NF-κB activation 41. YTHDF2 dictated degradation of *UBX domain protein 1* (*UBXN1*) mRNA through methyltransferase-like 3 (METTL3)-dependent m^6^A modification, which in turn activated NF-κB cascade 41. Chang *et al.,* also reported that METTL3 promoted the malignant progression of IDH-WT glioma possibly by enhancing NF-κB activation 40. Ji *et al.,* reported that elevated TRIM22 (tripartite motif 22) promoted GBM cell proliferation by activating NF-κB signaling 59.

Early studies have implied that Gαi2 could be important for NF-κB cascade activation. Conditional disruption of Gαi2 in CD11c^+^ DCs and MDSCs prevented NF-κB and STAT3 activation 22. Gαi2-depletion-induced NF-κB inactivation was possibly due to blocking IL-6 signaling 22. Under hepatic ischemia-reperfusion injury, increased Gαi2 expression promoted NF-κB pathway activation through phosphorylating mixed-lineage protein kinase 3 (MLK3) 60. In the present study, we found that Gαi2 was important for NFκB activation in glioma cells. Indeed, p65 phosphorylation, NFκB (p65) DNA-binding activity and expression of NFκB-dependent genes (*cIAP2* and *survivin*) were significantly decreased in Gαi2-depleted primary glioma cells, but were increased following Gαi2 overexpression. BAY-11-7082, the NFκB inhibitor, largely suppressed proliferation and migration of Gαi2-overexpressed P1 glioma cells. Importantly, decreased p65 phosphorylation was observed in subcutaneous and intracranial glioma xenografts with Gαi2 depletion. Therefore, promoting NFκB cascade activation should be one important mechanism of Gαi2-driven glioma cell growth.

Studies have proposed that Sp1 is an important transcription factor for the malignant progression of glioma. Yu *et al.,* found that Sp1 enhanced NLR family pyrin domain containing 6 (NLRP6) transcription to promote immune evasion, malignant behaviors and radio-resistance in glioma cells. Contrarily, Sp1 silencing suppressed* in vitro* glioma cell growth and tumorigenesis *in vivo* 61. Li *et al.,* reported that Sp1 upregulated the LncRNA LBX2-AS1 to promote proliferation and EMT in glioma cells 62. Tan *et al.,* discovered that miR-150-3p silenced Sp1 to hinder glioma cell growth 63. Our results supported that Sp1-dependent Gαi2 transcription was increased in glioma tissues and cells, which might be one primary mechanism of Gαi2 upregulation in glioma. In glioma cells Gαi2 expression was downregulated after Sp1 silencing, KO or inhibition. It was however increased following Sp1 overexpression. Therefore, the increase of Sp1-dependent transcription should be one key mechanism of Gαi2 overexpression in human glioma.

Here, TCGA LGGGBM cohorts were analyzed and *Gαi2*-asscoateid DEGs in glioma tissues were retrieved, including a significant number of genes with unknown functions in human glioma. Moreover, KEGG analyses showed that *Gαi2*-asscoateid DEGs were enriched in NFκB and other signaling cascades. Further studies will be needed to explore expression and potential functions of these *Gαi2*-asscoateid DEGs in glioma, and to test these enriched pathways in the progression of glioma. Their connection with *Gαi2* should also be analyzed.

## Conclusion

Together, overexpressed Gαi2 is important for glioma cell growth possibly by promoting NFκB cascade activation. Gαi2 is possibly a novel and promising therapeutic oncotarget of glioma.

## Supplementary Material

Supplementary figure.
