# Supplementary material for: G protein inhibitory α subunit 2 is a molecular oncotarget of human glioma
Source: Int J Biol Sci. 2023 Jan 9;19(3):865–79. doi: 10.7150/ijbs.79355 (PMC9909998; doi:10.7150/ijbs.79355)

Figure S1. The uncropped blotting images of the study.

Figure 2.

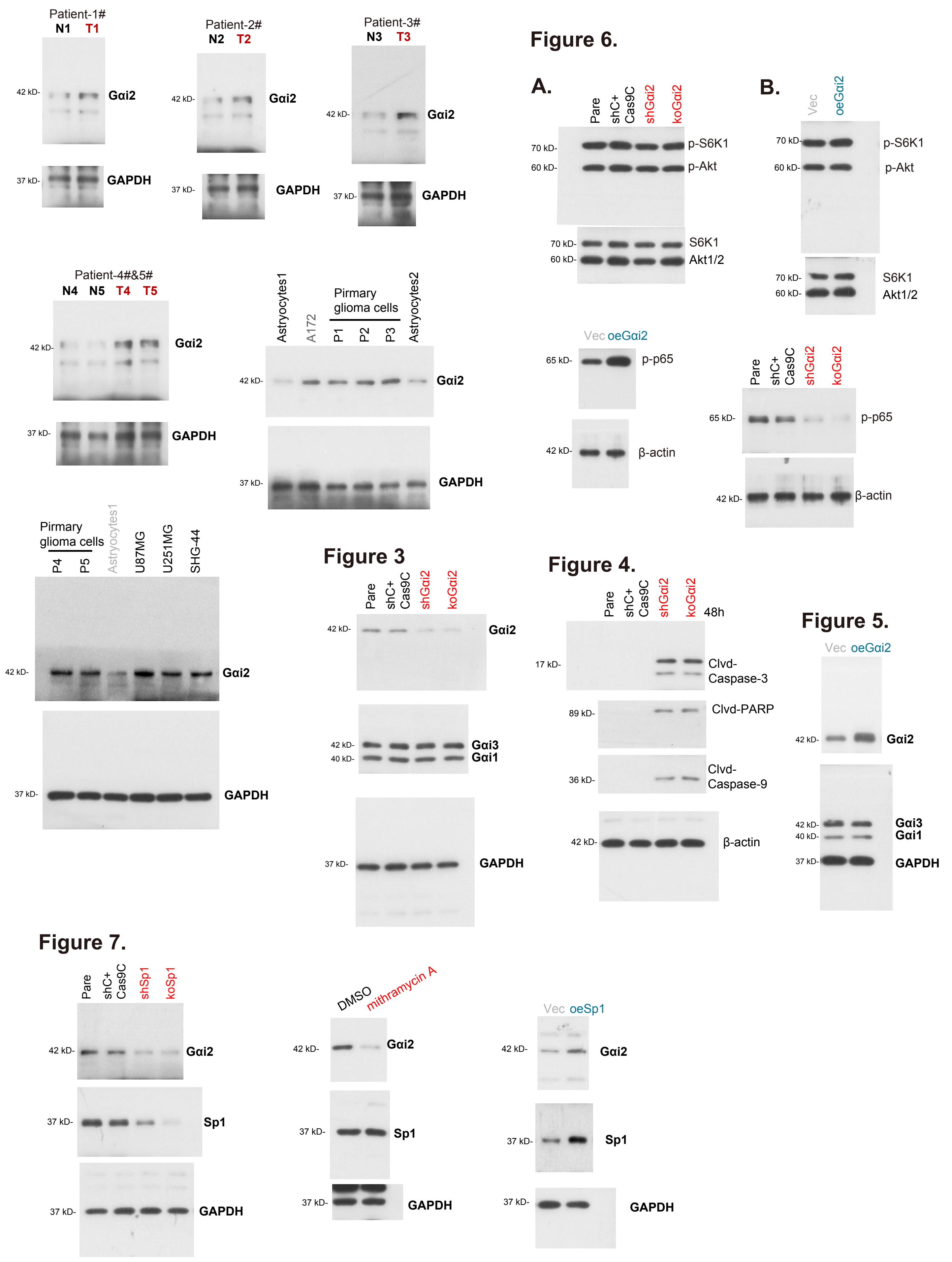

Figure 8.

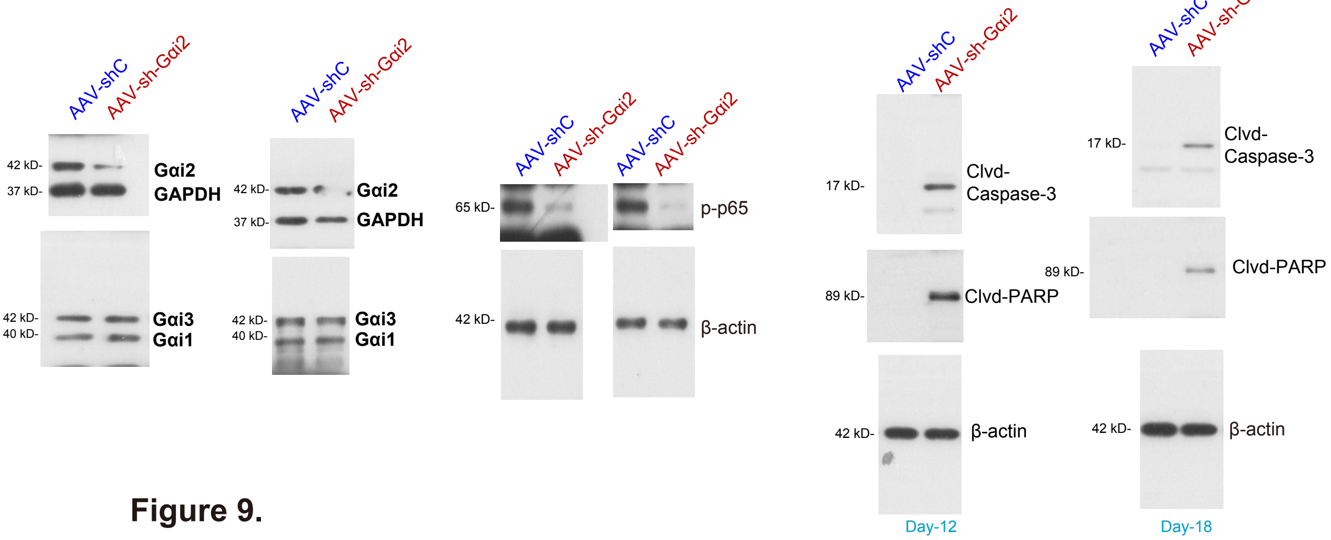

Figure 9.

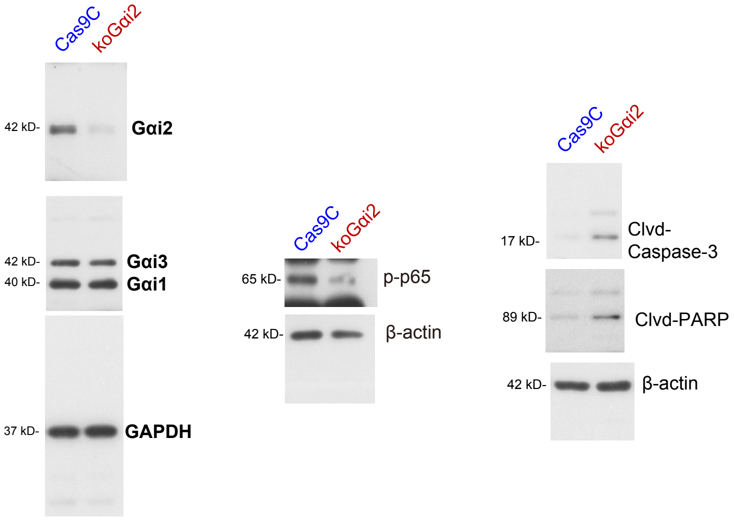

Supplement: Supplementary file 1 — Supplementary figure. [file ijbsv19p0865s1.pdf]
